# Simulations of Interdigitated Electrode Interactions with Gold Nanoparticles for Impedance-Based Biosensing Applications

**DOI:** 10.3390/s150922192

**Published:** 2015-09-02

**Authors:** Scott MacKay, Peter Hermansen, David Wishart, Jie Chen

**Affiliations:** 1 Electrical and Computer Engineering Department, University of Alberta, 116 St & 85 Ave, Edmonton, AB T6G 2R3, Canada; E-Mails: samackay@ualberta.ca (S.M.); phermans@ualberta.ca (P.H.); 2 Department of Computing Science, 2-21 Athabasca Hall, University of Alberta, Edmonton, AB T6G 2E8, Canada; E-Mail: david.wishart@ualberta.ca; 3 National Research Council/National Institute for Nanotechnology, 11421 Saskatchewan Dr NW, Edmonton, AB T6G 2M9, Canada

**Keywords:** COMSOL, biosensor, gold nanoparticles, interdigitated electrodes

## Abstract

In this paper, we describe a point-of-care biosensor design. The uniqueness of our design is in its capability for detecting a wide variety of target biomolecules and the simplicity of nanoparticle enhanced electrical detection. The electrical properties of interdigitated electrodes (IDEs) and the mechanism for gold nanoparticle-enhanced impedance-based biosensor systems based on these electrodes are simulated using COMSOL Multiphysics software. Understanding these properties and how they can be affected is vital in designing effective biosensor devices. Simulations were used to show electrical screening develop over time for IDEs in a salt solution, as well as the electric field between individual digits of electrodes. Using these simulations, it was observed that gold nanoparticles bound closely to IDEs can lower the electric field magnitude between the digits of the electrode. The simulations are also shown to be a useful design tool in optimizing sensor function. Various different conditions, such as electrode dimensions and background ion concentrations, are shown to have a significant impact on the simulations.

## 1. Introduction

Biosensor devices hold great potential for various applications in detecting the growth of cells and bacteria [1,2], detecting toxins [3], and detecting disease and monitoring disease progression [4,5]. Different detection designs based on optical, mechanical and electrochemical methods have been proposed [6,7,8]. These designs each have their own advantages and disadvantages. Electrical and electrochemical detection techniques are particularly well suited for quick and simple detection [8].

Although individual methods can vary, all electrical and electrochemical biosensors work by measuring changes in electrical properties caused by the presence of specific biological targets. These changes can be caused by interference in electric fields [9], chemical reactions [10], or from conductive labels [8]. Electrodes (usually gold) are used as interfaces both in applying electric fields to the tested samples and as a method of transmitting and measuring electrical detection signals. The types of measured electrical signal vary as well depending on the application, and can be simple impedance or resistance measurements [11], capacitance measurements [9], or electrical spectroscopy, taking measurements over a range of frequencies [10].

**Figure 1:**
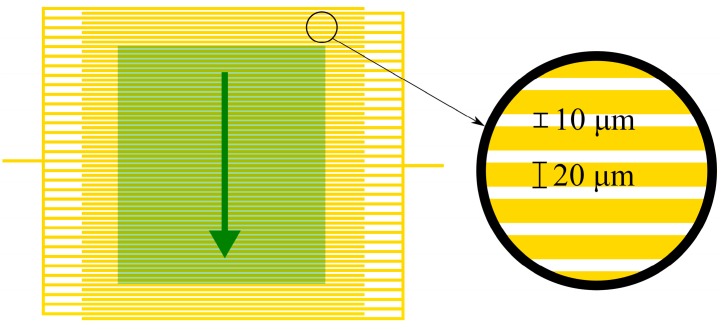
An example of gold IDE structure. The highlighted green region is the approximate region where a simulation would be valid. The green arrow denotes the cross sectional area that the 2D models in this paper simulate.

Beyond being a simple interface, the properties of the electrodes used in electrical and electrochemical biosensors can have a significant impact on the function and effectiveness of the resulting biosensor system. One prominent example of this is the use of interdigitated electrodes (IDEs) in biosensor systems. IDEs consist of two interlocking, but separated metal plates, with each having a number of individual digits which overlap with those of the other section (see Figure 1), essentially creating the same structure as microfabricated capacitors. Applying a voltage to IDEs, either AC or DC, creates an electric field between the digits. This electric field can be disrupted and altered by the presence of specific target biomolecules, cells, or electrically active labels and the resulting change can then be measured. It has therefore been possible use IDEs in a number of highly sensitive biosensing applications [1,2,12,13,14,15]. Nearly all biosensor applications of IDEs use gold as the electrode material, due to its biocompatibility.

Several key dimensions of IDEs can have a great impact on what particular application they are best suited for and how well they perform. Digit width and digit spacing are of particular importance, with smaller interdigit spacing generally resulting in more sensitive detection. Dimensions in the range of several micrometers (for both the digit width and spacing) have been shown to be effective for the detection of cells and bacteria [1], whereas electrodes with sub-micron dimensions have been made which are sensitive enough to detect DNA hybridization [13]. With such large differences in the dimensions required for different applications, the proper design and optimization of IDEs is essential for their use in effective biosensors.

Because a variety of different parameters and dimensions play a part in the resulting function of an IDE, it becomes impractical to design, manufacture and test all possible designs for a particular application. It is by far more practical to simulate the electric field and potential of IDEs using models. Simulations for the optimization of IDE dimensions for specific applications have previously been carried out. However, these simulations are specific to one particular application, such as for the detection of bacteria [1].

**Figure 2:**
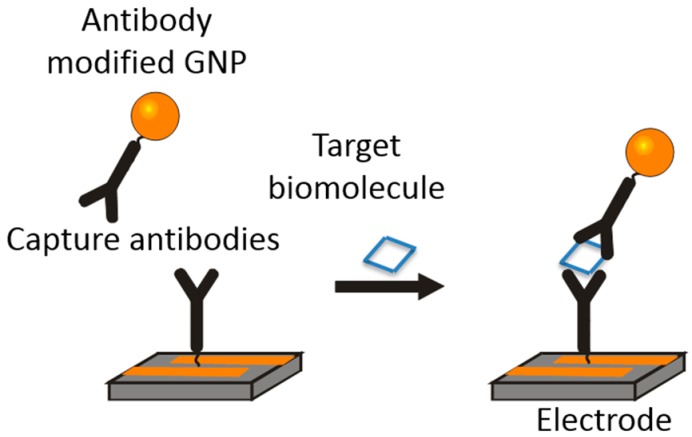
An example of the mechanism used for biomolecule detection. Here the target biomolecule allows for a connection between the modified GNP and electrode surface.

The specific purpose of the simulations presented in this paper is to find the optimum IDE dimensions and testing conditions for a gold nanoparticle enhanced impedance-based biosensor. This method uses aptamers or binding proteins, depending on the target biomolecule, to bind to specific biomolecules and allow for a connection between an electrode surface and modified gold nanoparticles. An example of this with antibodies is shown in Figure 2. Aptamers are single strands of DNA or polymers with structures that can be designed to bind specifically to target biomolecules [16,17,18,19]. In the case of the design presented here, detection is achieved by measuring changes in impedance caused by these attached gold nanoparticles. The presence of bound nanoparticles above the electrodes disrupts the electric field around the electrodes leading to decreased measured impedance. Initial experiments have shown that this method is effective for detecting a variety of target biomolecules, however, optimization of both the electrode dimensions and testing conditions is still required to create a fully realized biosensor device using this technique.

As mentioned previously, a number of different factors contribute to the exact conditions required for optimal detection. In the case of impedance-based detection, the most important factor is the electric field generated at the electrode and how this can be best influenced for effective detection. These factors include the digit width, spacing and height in the IDE, applied voltage to the electrode and the particular background solution used for testing. Since, as in many biosensor applications, measurement here takes place in an aqueous environment, it is very important that this background solution be controlled. This is so that the solution does not interfere with measurements, does not interfere with the target biomolecule or recognition elements, and does not interfere negatively with the electrode. Some interference with the electric field in the system is actually beneficial in making a more sensitive biosensor as the ionic concentration of a background solution can establish an electrical screening layer that, with the right design, can isolate the electric field to only the region above the electrode where binding and detection occurs, increasing sensitivity.

The goal of this article is to use COMSOL Multiphysics simulations to show the effect of using gold nanoparticles on IDE systems for impedance-based biosensing techniques. Furthermore, these simulations can be used to find optimal design conditions to achieve the highest sensitivity.

## 2. COMSOL Simulations

A simulation for the electrical properties of IDEs was carried out using COMSOL Multiphysics software. This finite element analysis software was used to model various geometries and conditions for IDEs and is able to display several electrical properties of such systems, including time dependent properties, such as effects of changing ion concentrations.

For simulations of electrical screening above IDEs, a Nernst-Poisson method was used. The overall charge distribution of the system is determined by the Poisson equation:

(1) $$\nabla^{2}\phi =-\frac{\rho }{\varepsilon }$$

where *ϕ* is the electrical potential of the system, *ε* is the electric permittivity of the solution and *ρ* is the charge density caused by the ions in solution. The charge density is also related to the concentration of each type of ion in the solution:

(2) $$\rho =eN_{A}\sum c_{i}z_{i}$$

here the concentration of each ion (*c_i_*) refers to the concentration as a function of location in the system, *e* is the elementary charge, *N_A_* is Avogadro’s number and *z_i_* is the charge number (+1 or −1 for singly charged ions). Although the initial condition is for a uniform distribution of ions, they drift due to the applied voltage, causing variations in the overall concentration. These changes in the concentration (*c_i_*) of the ions over time (*t*) is described by the Nernst-Planck equation:

(3) $$\frac{\delta c_{i}}{\delta t}=\nabla \left[D_{i}\nabla c_{i}-uc_{i}+\frac{D_{i}z_{i}e}{k_{B}T}c_{i}\left(\nabla \phi +\frac{\delta A}{\delta t}\right)\right]$$

where *D* is the diffusivity of the ions, *u* is the fluid velocity, *k_B_* is the Boltzmann constant, *T* is the temperature and *A* is the magnetic vector potential. This equation is simplified in this case as the fluid itself does not move (*u* = 0) and there are no magnetic fields (*A* = 0):

(4) $$\frac{\delta c_{i}}{\delta t}=\nabla \left[D_{i}\nabla c_{i}\frac{D_{i}z_{i}e}{k_{B}T}c_{i}\nabla \phi \right]$$

which accounts for both ion migration due to the applied voltage at electrodes ($\frac{D_{i}z_{i}e}{k_{B}T}c_{i}\nabla \phi$), and due to the concentration gradient created by this applied voltage ($D_{i}\nabla c_{i}$). Using these above equations, the COMSOL simulations are able to calculate the changes in concentrations of ions in IDE systems over time, as well as the resulting changes in electric field and electric potential distributions.

The actual model of the electrode system constructed in COMSOL consists of three basic parts: gold digits, a water background and ion concentrations. Taking advantage of the symmetry of the system along the axis of the digits, to add simplicity, these models are based on 2D planes, which are then extended into 3D. Therefore, all of the constructed digits and the water background were constructed as rectangles in one plane then extended to form the final model. To reduce complexity and to save time, only a small number of digits were modeled, and to avoid discrepancies, results for the simulations were taken only for the middle of these simulated sections. The same applies to the simulated gold nanoparticles. They consist of 2D circles placed above the electrodes, which become 3D when the simulation is extended. This results in “nano-cylinders” rather than perfect spheres. By limiting the depth of the extension however, these simulations can still study the effect of small amounts of gold (the nanoparticles) bound to the electrodes, while keeping the overall system simple, simulation mesh sizes fine enough for accurate results and still keep computation time reasonable. The material properties of the gold electrodes and surrounding water were added to the model for the various diffusion and electrical properties necessary. To model the ionic concentration, two entities (for positive and negative ions) were added with equal concentrations, for overall neutrality. The properties of the ions can be defined in the software, including diffusion constants, mobility and charge. The space charge density in the water is also defined using the varying concentrations of the ions. A free triangular mesh is used with this model. The maximum element size is specified to be 3.31 µm and the minimum is specified to be 18.8 nm. Along the surface of each digit and each gap the number of elements is fixed to 100. This in addition with a maximum element growth rate of 1.3 allows for a much greater mesh resolution around electrodes. Element size on other boundaries in the model is dictated by COMSOL’s physics controlled mesh.

### 2.1. Electric Potential and Electric Screening Simulations

**Figure 3:**
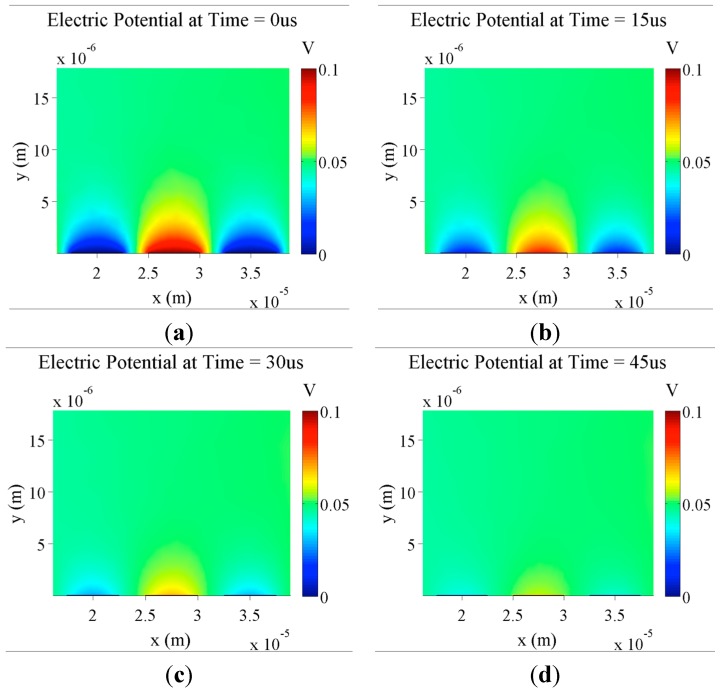
Simulation of the formation of electrical screening layers over time. Plots show the electric potential of IDEs (5 µm digit width and 2.5 µm spacing) with 0.1 V DC applied with a background KCl concentration of 150 µM. Plots are taken at five different time points: (**a**) *t* = 0 s; (**b**) *t* = 15 µs; (**c**) *t* = 30 µs; (**d**) *t* = 45 µs.

First, the electrical potential of IDEs was simulated using COMSOL software. This simulation also tracks the changing ion concentrations over time, making it possible to show the electric screening process over the electrode. Electric screening occurs when mobile ions in solution migrate due to the electric potential in the system and this mobile charge accumulation cancels out the electric field of the electrodes over a set distance as seen in Figure 3.

Since the final intent is for there to be an AC voltage applied to the electrodes, it is important to determine how quickly electrical screening is established. An AC signal is required to determine the impedance of the electrodes, as there are both capacitive and resistive contributions in an IDE system. Figure 3 shows that with 0.1 V DC applied to electrodes and a KCl concentration of 150 µM in solution, full electrical screening is established within 50 µs. This establishment time will vary depending on the exact ion species used and the concentration. In these simulations, KCl is used because it has singly charged ions and well known migration constants for the simplest simulations for a salt solution. For this and other simulations presented here, an ion concentration of 150 µM is used. This value is based on early tests on fabricated electrodes with different buffer solutions. A solution with 150 µM KCl was found to give the optimal baseline impedance for these electrodes, with less concentrated making the resulting impedance too capacitive and much higher being too conductive as to just measure the resistance of the solution rather than the total impedance of the electrodes. In a later section, the effects of varying the ion concentration in simulations are investigated.

For screening when AC voltages are applies, it will be essential for full screening to be established in less time than the period of the AC voltage in order to have a consistent detection environment. Some simulations were done using a time dependent sinusoidal applied voltage at various frequencies. However, the evolution of these simulations over time provided no extra insight into the function of the sensor system. In this case, for example, an establishment time of 50 µs corresponds to a maximum AC frequency of 20 kHz. Since measurements in the final biosensor design are intended to be at lower frequencies (1–10 kHz), there is therefore enough time in each cycle for screening to be established.

For the simulations presented in this paper, only DC voltages are applied, and to ensure stable ion distributions, results are shown after an excessively long time period (100 ms). This ensures that any results are not influenced by transient properties of the environment. In actuality, it would take much less time for screening to be established.

### 2.2. Electric Field Simulations for Different Electrode Dimensions

**Figure 4:**
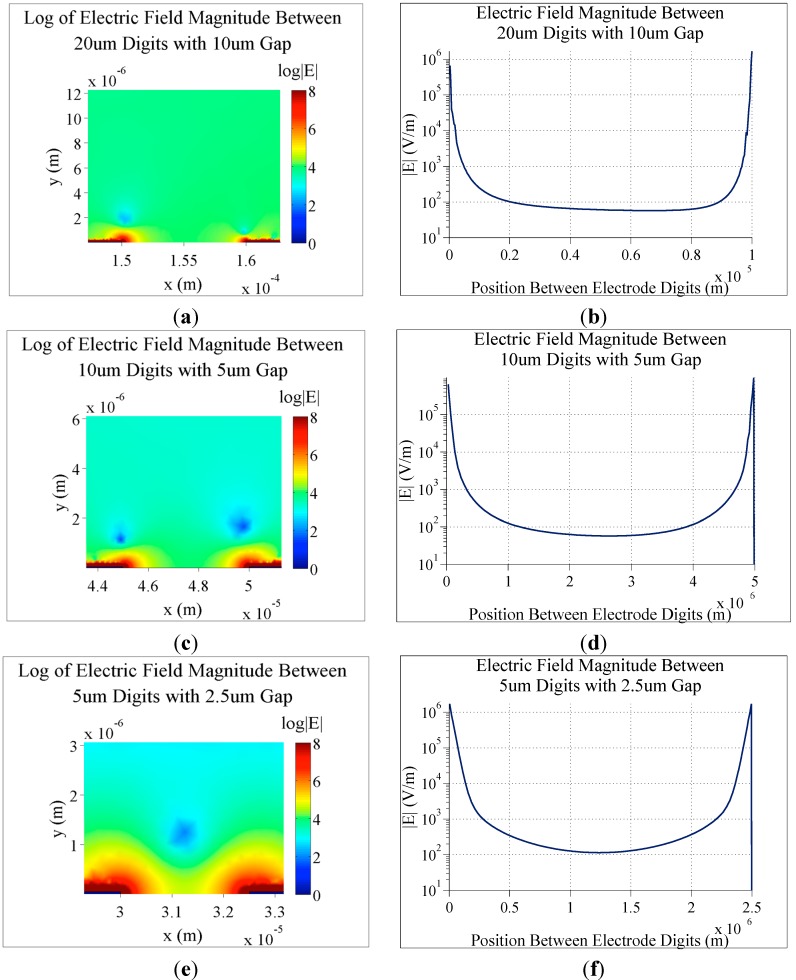
Simulations of the electric field magnitude for three different electrode configurations. In all cases, 0.1 V DC is applied to the electrodes, there is a background KCl concentration of 150 µM and simulations are shown after electric screening has developed. (**a**) Log of the electric field magnitude for 20 µm digits with 10 µm spacing; (**b**) The electric field magnitude between two of the digits from (**a**); (**c**) Log of the electric field magnitude for 10 µm digits with 5 µm spacing; (**d**) The electric field magnitude between two of the digits from (**c**); (**e**) Log of the electric field magnitude for 5 µm digits with 2.5 µm spacing; (**f**) The electric field magnitude between two of the digits from (**e**).

These COMSOL simulations also make it possible to plot the electric field between individual electrode digits. This is particularly useful when trying to determine the ideal dimensions for digit width and spacing.

As seen in the simulations in Figure 4, as the spacing between digits is increased, the resulting electric field magnitude between them decreases. This electric field between digits is of particular importance when considering biosensor design, as disruptions in this electric field caused by specific binding of target biomolecules are the basis for many electrical biosensor designs. For example, bacteria on the surface of IDEs can disrupt this electric field, resulting in an increased impedance measured across the electrode [1]. In the same way, conducting tags can cause decreased impedance.

### 2.3. Impact of Gold Nanoparticles on Electric Field Magnitude

The mechanism of detection in the proposed biosensor is for gold nanoparticles (GNPs) bound to the IDEs to affect the electric field of the system and result in a measurable impedance change. The effect of nanoparticles near the surface of the electrodes can be modeled in COMSOL as well by using gold circles and thinner “extensions” of the 2D plane. The following figures show that a distribution of GNPs placed above the surface of the gold digits can have an effect on the electric field measured directly between the electrode digits (measured from the middle of the of the sides of the digits). GNPs in general decrease the electric field magnitude directly between digits, which would result in a lower measured impedance for the overall electrode than a measurement taken without GNPs present.

**Figure 5:**
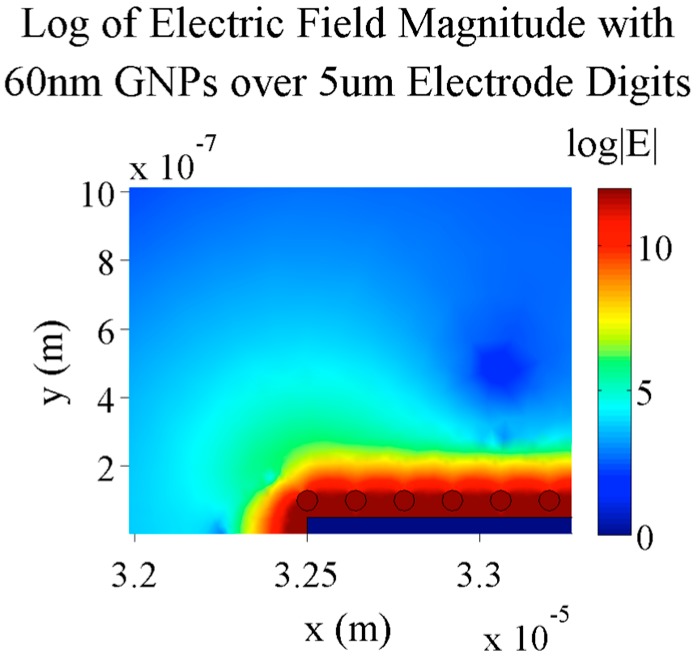
Electric field magnitude of a 5 µm gold electrode with 60 nm GNPs placed 20 nm above the electrode surface after 0.1 s to completely establish electric screening. A voltage of 0.1 V is applied to the electrode here and the field is in the presence of a 150 µM KCl buffer solution.

**Figure 6:**
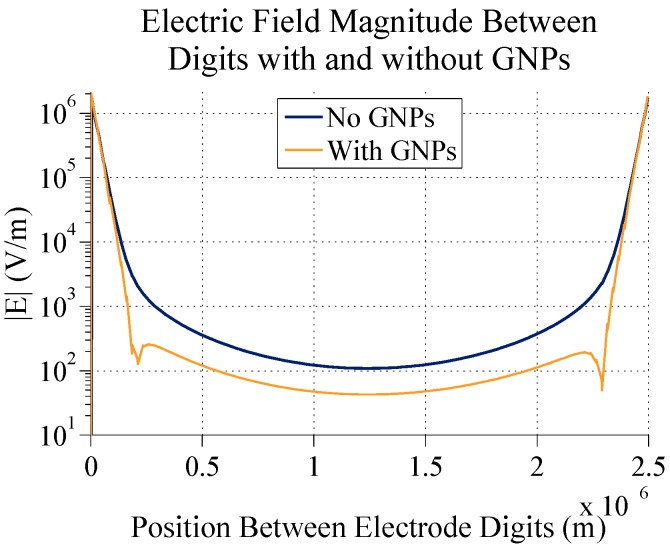
A graph comparing the electric field magnitude between 5 µm digits with 2.5 µm spacing with and without 60 nm gold nanoparticles 20 nm above the electrodes.

Figure 5 and Figure 6 show that GNPs on top of electrode digits do change the profile and decrease the electric field magnitude directly between digits. In the case shown here, at the middle of the interdigit space, there is a difference of about 66.5 V/m in the electric field magnitude. This demonstrates how gold nanoparticles can indeed be used to measure detection of targets bound to the surface of IDEs. Here, and in further simulations, 60 nm GNPs are used for bound nanoparticles. This diameter was chosen mainly to eventually compare simulation results with actual tests (for which 60 nm GNPs will be used) and to keep results in line with work that had been previously done with attaching biomolecules to 60 nm GNPs. In a later section, different sizes of GNPs will be simulated.

Many different factors contribute to the overall effectiveness of IDE systems for biosensor applications. By adjusting these various factors, the electric field-altering effect of GNPs can be improved to make the most effective sensor possible.

#### 2.3.1. Impact of Ion Concentration on GNP Effect

In the previous simulation, the background of the electrode system was set as water containing 150 µM of KCl. However, changing this value can have a great impact on the entire system and the effect that GNPs have. Changing the ion concentration changes the amount of electrical screening around the electrodes and also the electrical properties (such as conductivity) between the electrodes.

**Figure 7:**
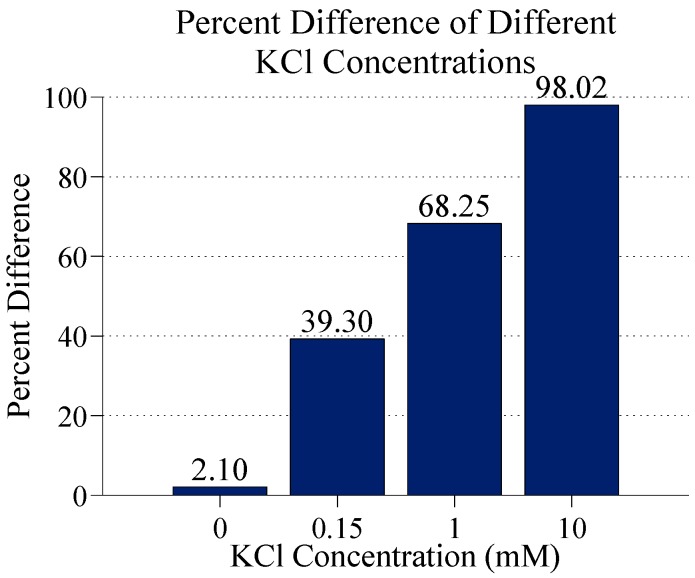
The percent difference in the electric field magnitude at the center of the gap between electrodes digits with and without 60 nm GNPs at different KCl ion concentrations. GNPs are 20 nm above electrodes, electrodes have 5 µm digits with 2.5 µm gap space and 0.1 V applied voltage.

**Figure 8:**
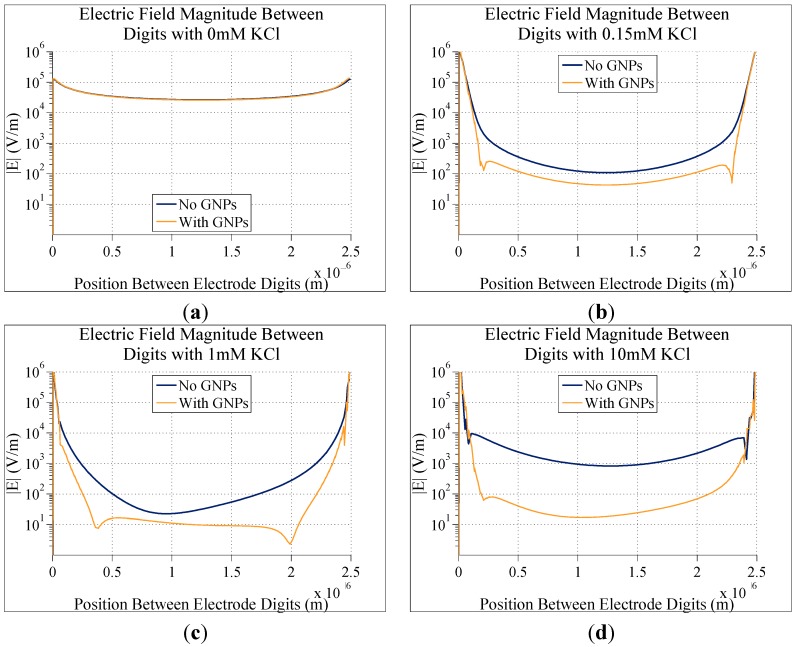
Graphs comparing the simulated electric field magnitude directly between two digits of electrodes with and without bound gold nanoparticles. In all cases, electrodes consisted of 5 µm digits with 2.5 µm gaps between digits, and 0.1 V DC applied to them. The only difference between each graph is the concentration of KCl in the solution in the electrodes: (**a**) 0 M; (**b**) 0.15 mM; (**c**) 1 mM; and (**d**) 10 mM.

In order to investigate the effect of ion concentrations four different ion concentrations were simulated on electrodes with 5 µm digits with 2.5 µm electrode gaps. With no ions (just water) there is almost no difference in the electric field magnitude with and without GNPs. As the ion concentration is increased, both the electric field magnitude with and without bound GNPs decreases, but the electric field magnitude between electrodes with GNPs decreases by a greater amount. The percent difference between electric field magnitudes at the center of the gap (half way between electrode edges) for bare and GNP bound electrodes for different ion concentrations is shown in Figure 7 and graphs of the electric field in these cases is shown in Figure 8.

The percent difference in the electric field magnitude increases from nearly 40% at 0.15 mM KCl to nearly 100% at 10 mM. Although the difference in electric field magnitude increases as ion concentration increases, there are practical limits to how high this can go. Ion concentrations that are too high could interfere with biological elements in the biosensor system and potentially disrupt GNP bonding. Additionally, increasing the ion concentration too high causes too much conduction between electrode digits, such that the conductivity of the ion solution overwhelms any effect of the nanoparticles on the impedance of the system. Therefore, it is necessary to find a balance between having an ion concentration, which is high enough to enhance detection, but still low enough as to not drown out the effects of bound nanoparticles.

#### 2.3.2. Impact of Electrode Dimensions on GNP Effect

As shown previously, the dimensions of the electrode system, particularly the spacing between digits, can have a significant effect on the electric field between the digits. Therefore changes in electrode dimensions should also change the effect of GNPs on the electrodes. In Figure 7 above, 5 µm digits with 2.5 µm spacing was used for the three ion concentrations tested. This particular set of dimensions had the most uniform electric field profile, but different dimensions can also be tested.

**Figure 9:**
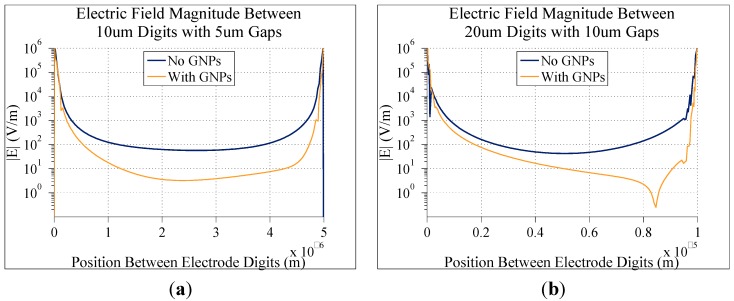
Graphs comparing the simulated electric field magnitude directly between two digits of electrodes with and without bound gold nanoparticles. In all cases, there was a background concentration of 10 mM KCl, and 0.1 V DC applied to the electrodes. The only differences between each graph are the dimensions of the electrodes: (**a**) 10 µm digits with 5 µm gaps; (**b**) 20 µm digits with 10 µm gaps.

Figure 9 shows the electric field magnitudes with and without bound GNPs (60 nm diameter, bound 20 nm above the electrodes) for two additional electrode configurations (with 0.1 V DC applied between electrodes and 10 mM KCl ion concentration in the background solution) with larger digits and gaps. The overall electric field magnitude decreases in all cases when the gap and digits increase. In Figure 9b, with 20 µm digits with 10 µm gaps, there is a non-symmetric dip in the electric field on the right side of the gap. This is likely due to the positioning of the nanoparticles on the electrode causing a lack of symmetry. The exact placement of nanoparticles obviously cannot be controlled in a practical system, so here for comparisons values of electric field magnitude are taken at the exact center of the gaps.

**Figure 10:**
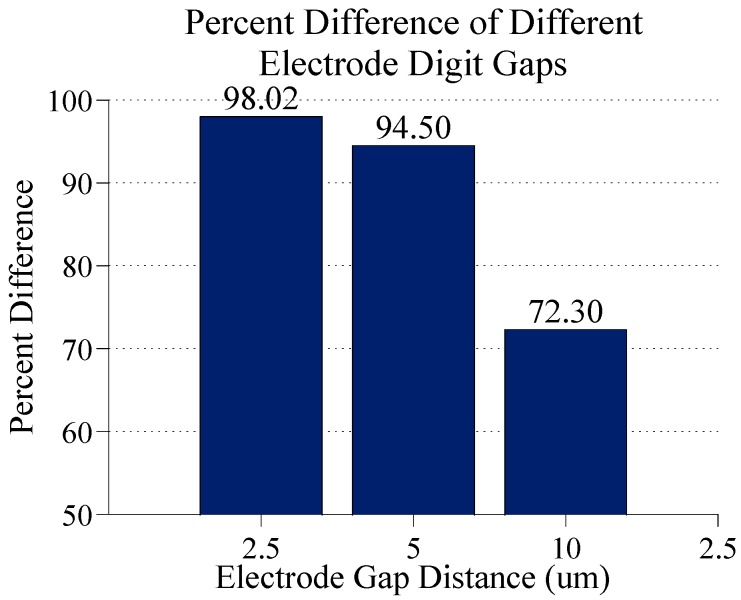
The percent difference in the electric field magnitude at the center of the gap between electrodes digits with and without 60 nm GNPs bound to the surface of the electrodes with varying electrode dimensions. GNPs are 20 nm above electrodes, 10 mM KCl concentration and 0.1 V DC applied to the electrodes.

Figure 10 shows a comparison of the percent differences in the electric field magnitudes at the center of the gap between digits for the different electrode geometries. In all cases, there is a 2:1 ratio in digit width to gap width. As seen here, doubling the gap distance from 2.5 µm to 5 µm results in a slight decrease in the percent difference in electric field magnitude caused by bound GNPs. Doubling the gap again to 10 µm causes a significant decrease in this difference, down to 72.3%. These results show that the smaller the electrode dimensions, the more sensitive the model is to bound GNPs. This yields overall better result. This makes physical sense as well. The closer together the digits of the electrode are, the more confined the electric field becomes, like a parallel plate capacitor. Therefore, effects of disruptions in these electric fields are more pronounced. Conversely, the electric fields between larger gaps will be less sensitive, making them better suited for detecting larger species, such as attached cells or bacteria. Therefore for this application electrodes should be designed to have the smallest dimensions possible, but the benefit of smaller dimensions becomes less significant as the dimensions decrease. Electrodes with slightly larger dimensions could still be nearly as effective without having to resort to more expensive and specialized microfabrication techniques to yield the absolute smallest dimensions possible.

#### 2.3.3. Impact of GNP Size and Placement

For the above simulations, the properties and positions of GNPs themselves were kept constant, however, these exact conditions may not be possible. For example, the linkers between the electrodes and nanoparticles may be larger. Commonly used biological recognition elements, such as proteins and aptamers, can vary in size greatly, and extra molecules and modifications are sometimes necessary to facilitate bonding to the electrodes or nanoparticles.

**Figure 11:**
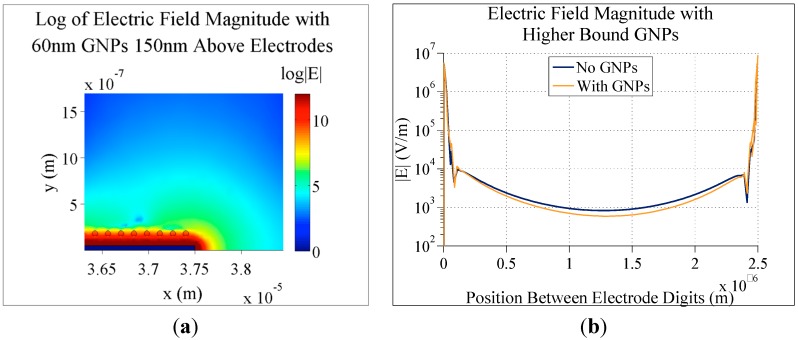
(**a**) Simulation of the electric field magnitude of 5 µm digit and 2.5 µm gap electrodes with 0.1 V applied, 10 mM KCl concentration and 60 nm GNPs bound 150 nm above the electrodes; (**b**) A graph of the electric field magnitude directly between the gaps in these electrodes with and without gold nanoparticles.

Figure 11a shows that, if the binding molecules attaching GNPs to the electrode are too long, the nanoparticles will be outside of the electrical screening distance of the biosensor system. This results in a negligible difference in electric field magnitude with and without nanoparticles (only 2.7% difference at the center of the gap). The screening depth of the system can be adjusted by changing the background ion concentration, and potentially change the effect of bound nanoparticles.

The size of gold nanoparticle used could also be influenced by the individual applications, as different modifications to nanoparticles are necessary for different biosensor applications (e.g., different attachment molecules required to bind to nanoparticles).

**Figure 12:**
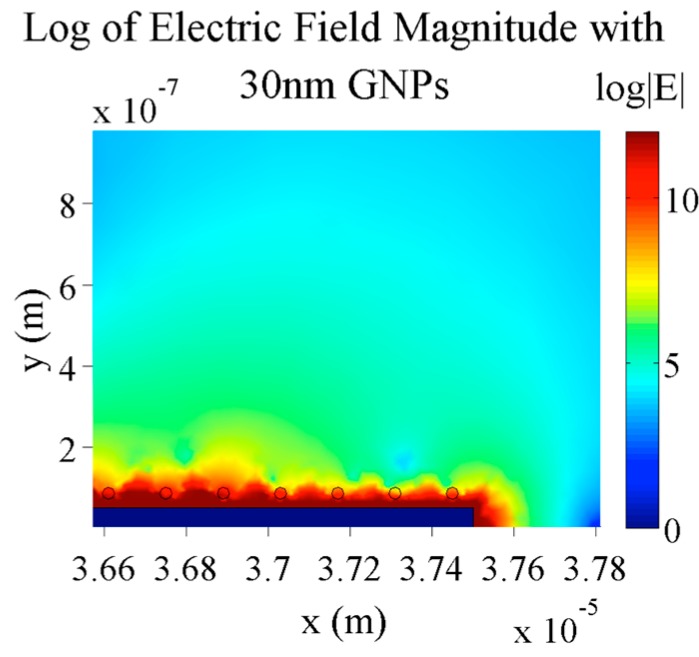
Simulation of the electric field magnitude of 5 µm digit and 2.5 µm gap electrodes with 0.1 V applied, 10 mM KCl concentration and 30 nm GNPs bound to the electrodes.

With smaller nanoparticles (30 nm diameter), as shown in Figure 12, there is actually very little change in the electric field magnitude difference between electrode digits. The percent difference in this simulation is 98.5% compared to 98.0% for the same conditions and 60 nm nanoparticles. In both cases, there were the same number of nanoparticles on the electrodes, only the size of the nanoparticles was different.

**Figure 13:**
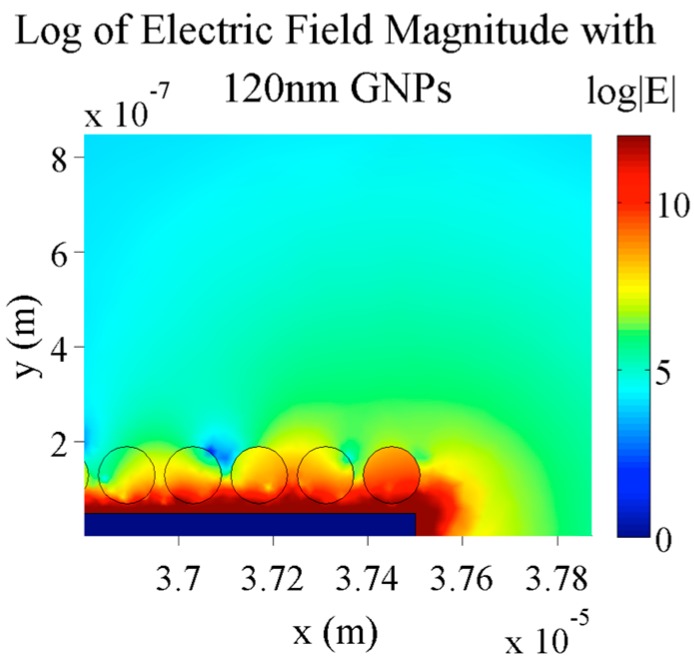
Simulation of the electric field magnitude of 5 µm digit and 2.5 µm gap electrodes with 0.1 V applied, 10 mM KCl concentration and 120 nm GNPs bound to the electrodes.

With larger nanoparticles (120 nm diameter), shown in Figure 13, there is still no significant difference in the electric field magnitude percent difference. Actually, in this particular case, the difference at the center of the gap is slightly lower than with smaller nanoparticles at 94.3%.

This indifference to the size of the nanoparticles is most likely due to the electrical screening layer established over the electrode. In each case, regardless of the size of the nanoparticles, the amount of gold in the areas over the electrodes that actually influence the electric field in the gaps stays approximately constant. As seen in Figure 12 and Figure 13, almost all of the small nanoparticles are within the red region above the electrode whereas only a small portion of the large nanoparticles is in this region.

Since there is no major difference in these three cases at this fixed geometry and ion concentration, different criteria should be used when determining ideal nanoparticle size. Smaller GNPs could be advantageous for better stability in solution and better packing efficiency when bound to electrode surfaces, whereas larger nanoparticles can be modified with more binding molecules for better binding efficiency.

## 3. Discussion

Although these simulations have been shown to be powerful tools in showing the mechanism and some of the optimal conditions for using IDEs in biosensor applications, there are currently some limitations to these simulations. The simulations presented here only consider a DC applied voltage for the electrodes, however, for impedance measurements, AC voltages must be applied in the final design. This could affect ion transport and screening depths, but since these simulations are taken is very short time frames, DC voltages can be considered good approximations conditions for most of AC cycles. Additionally, the effects of actual biological molecules are not considered in these simulations. These molecules, either in solution around the electrodes or chemically bound to the surface of the electrodes and GNPs, could potentially also interact with the electric field as many biological molecules contain charges and can bind to certain surfaces. The goal of these simulations is to give general design rules to create the best overall system regardless of the final biomolecules involved. The versatility of this system means that many different kinds of biorecognition elements and biological samples can be involved in the detection process, with each having potential effects, positive or negative, on the function of the sensor. Because of these complexities, these potential effects were not considered in simulations. Similarly, additional fluid dynamic and electrophoretic effects were not considered in simulations. Given the dimensions and applied voltages and ion concentration ranges considered, these effects should be minimal compared to the factors considered. Other groups have done similar simulations on IDE biosensors for detecting cells and bacteria [1,20], and for simulations involving other electrode configurations [21,22]. The uniqueness in the simulations presented here comes from the specific application using nanoparticle to reduce the measured impedance across electrodes and in using these simulations as a design tool in optimizing sensor function.

Future work will focus on designing actual IDEs to validate the results of these simulations. Results of studies of actual biosensor and electrode systems can assist in choosing future parameter for further simulations and electrode optimization. Additionally, to make these simulations more comparable with actual device results, it will be necessary to simulate actual impedance values for these systems, rather than just trends in electric field differences between individual electrode digits to evaluate different models. Further optimization of electrode dimensions and conditions is also required. Although general trends have been established here for electrode digit spacing, ion concentration and GNP properties, optimizing all of these properties and more simultaneously will require more extensive simulations.

## 4. Conclusions

A method for simulating impedance-based IDE biosensor systems was developed using COMSOL Multiphysics software. These simulations can be used to show the effects of ions in solution over time in the establishment of electric screening layers above electrodes as well as the electric fields between the digits of the electrodes. The effect of bound gold nanoparticles under different conditions was also simulated. Under certain conditions, smaller digits and interdigit gap spacing, and higher background ion concentrations in general lead to higher percent differences in the electric field magnitude between digits, corresponding to lower measured impedance. Although smaller digits and interdigit gap spacing resulted in higher percent differences, the benefit of smaller dimensions diminishes as dimensions decrease. This indicates that electrodes with very small dimensions are not much more effective than slightly larger electrodes which do not require expensive fabrication techniques. Similarly, while higher background ion concentrations result in higher percent differences, too high would overwhelm the effect of GNPs on the system. Simulations showed that GNPs too far away from the electrodes and outside of the established electric screening distance had very little effect on the electric field between digits. The size of the nanoparticles in the simulations did not greatly affect the results. Future work with these simulations will be focused on further optimization of conditions to maximize results and make the simulations more in line with real-world devices and comparisons between simulated and actual device results. These simulations can be used as a tool for designing effective biosensor devices. The validity and effectiveness of these simulations will be evaluated by comparison to actual results from fabricated devices as well.
